# Immune Thrombocytopenia in Two Unrelated Fanconi Anemia Patients – A Mere Coincidence?

**DOI:** 10.3389/fped.2015.00050

**Published:** 2015-06-08

**Authors:** Anna Karastaneva, Sofia Lanz, Angela Wawer, Uta Behrends, Detlev Schindler, Ralf Dietrich, Stefan Burdach, Christian Urban, Martin Benesch, Markus G. Seidel

**Affiliations:** ^1^Division of Pediatric Hematology/Oncology, Department of Pediatrics and Adolescent Medicine, Medical University Graz, Graz, Austria; ^2^Division of Pediatric Hematology/Oncology, Department of Pediatrics, Technische Universität München, Munich, Germany; ^3^Institute of Human Genetics, Biozentrum, University of Würzburg, Würzburg, Germany; ^4^German Fanconi Anemia Support Group, Unna, Germany

**Keywords:** immune thrombocytopenia, Fanconi anemia, bone marrow failure syndrome, DNA repair defect, Evans syndrome, danazol, FANCA, FANCD2

## Abstract

Thrombocytopenia and pancytopenia, occurring in patients with Fanconi anemia (FA), are interpreted either as progression to bone marrow failure or as developing myelodysplasia. On the other hand, immune thrombocytopenia (ITP) represents an acquired and often self-limiting benign hematologic disorder, associated with peripheral, immune-mediated, platelet destruction requiring different management modalities than those used in congenital bone marrow failure syndromes, including FA. Here, we describe the clinical course of two independent FA patients with atypical – namely immune – thrombocytopenia. While in one patient belonging to complementation group FA-A, the ITP started at 17 months of age and showed a chronically persisting course with severe purpura, responding well to intravenous immunoglobulins (IVIG) and later also danazol, a synthetic androgen, the other patient (of complementation group FA-D2) had a self-limiting course that resolved after one administration of IVIG. No cytogenetic aberrations or bone marrow abnormalities other than FA-typical mild dysplasia were detected. Our data show that acute and chronic ITP may occur in FA patients and impose individual diagnostic and therapeutic challenges in this rare congenital bone marrow failure/tumor predisposition syndrome. The management and a potential context of immune pathogenesis with the underlying marrow disorder are discussed.

## Introduction

Thrombocytopenia and pancytopenia are frequent hematologic manifestations of Fanconi anemia (FA) ascribed to a varying, increasing degree of congenital bone marrow failure and developing myelodysplasia. Treatment options range from none (observation) to therapeutic administration of androgens and, in case of transfusion dependence or signs of (pre-) malignancy, e.g., cytogenetic aberrations indicative of clonal evolution, hematopoietic stem cell transplantation. At least 17 genes are known to be involved in the pathogenesis of FA. Stringent genotype–phenotype correlations are rare in classical FA and have consistently only been observed for variant groups, e.g., FA-D1 and FA-N (1–3).

Immune thrombocytopenia (ITP) is observed as an acquired acute and self-limited benign post- or para-infectious or idiopathic cytopenia in otherwise healthy children that, if treatment is required, usually responds well to high-dose intravenous immunoglobulins (IVIG) and/or corticosteroids (4). Chronic ITP may develop and indicate an underlying immune hematologic disorder (5). Refractory chronic ITP with bleeding diathesis sometimes requires additional immunosuppressive or thrombopoietin agonist treatment, or splenectomy. We observed the clinical course in two patients with thrombocytopenia of immune origin in the context of FA. Here, we delineate the diagnostic and therapeutic challenges of this previously unnoticed concurrence and discuss potential implications.

## Patients and Methods

Two patients who have been diagnosed, treated, and prospectively monitored for FA at pediatric hematology/oncology departments at the Medical University Graz and the Technische Universität München (TUM) were found to suffer from ITP in 2014. The data presented here were obtained by retrospective chart review. Laboratory tests were performed according to clinical needs and routine standard procedures. After mitomycin C-mediated induction of G2 arrest and chromosomal breaks and/or complementation group analysis for FA, genetic testing was performed by means of multiplex ligation-dependent probe amplification (MLPA) and exon-scanning sequencing of genomic DNA of all exons of *FANCA* (patient 1) and of *FANCD2* (patient 2). Both patients are registered within the German registry for FA FAR01 of the German Society for Paediatric Oncology and Haematology (GPOH). The present study was performed upon informed consent in accordance with Declaration of Helsinki and approval of the responsible internal review boards.

## Results

### Patient report 1

**Figure 1:**
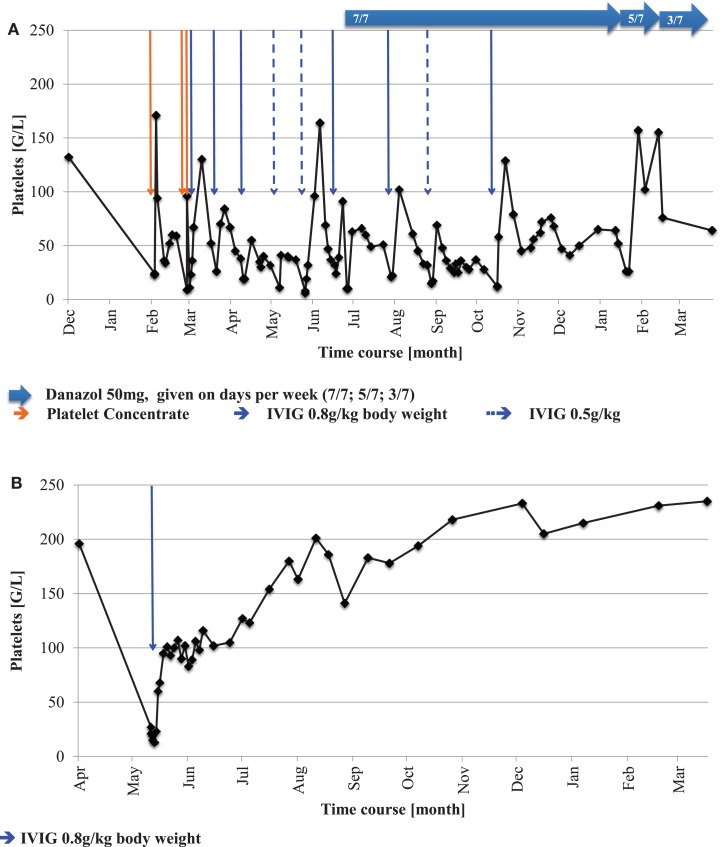
**Platelet count and treatment of two girls with ITP and FA over time**.

The first patient is a currently 3-year-old female, who was term-born and small for gestational age. Additionally to intrauterine growth retardation, other stigmata consistent with FA were present (Table 1). Diagnosis was confirmed *in vitro* by typical diepoxybutane- and mitomycin c-induced double strand break induction, G2 arrest, and a *FANCA* mutation (EX2_6del heterozygous, second mutation yet elusive). Esophageal atresia type IIIB required repetitive dilatation, until surgical intervention (Nissen fundoplication) at 17 months of age was undertaken. Prior to surgery, peripheral blood counts were stable within normal ranges. Baseline bone marrow evaluation had not been performed. Postoperatively, an isolated mild thrombocytopenia (minimum 70,000/μl) was observed. Platelet counts recovered spontaneously to near normal ranges (>100,000/μl) within the next months. A second abrupt and more pronounced platelet decline (23,000/μl) along with generalized petechial exanthema occurred 4 months later following anesthesia for an esophageal passage imaging study. Response to platelet transfusions was only transitory (Figure 1A), and repeated platelet transfusions were given to control the purpura. However, a brief increase of platelet numbers was always followed by a rapid decline (Figure 1A). An evaluation for allogeneic stem cell transplantation and a donor search were initiated.

**Table 1:** **Patient characteristics of two girls with FA and ITP**.

	Patient 1		Patient 2
	3 years, female		7 years, female
**Genotype**			
	*FANCA* compound heterozygous: EX2_6del; 2nd yet elusive		*FANCD2* compound heterozygous: 3-bp-deletion; missense substitution at codon 815
**Clinical presentation at birth and at diagnosis of FA**			
Pregnancy [week]	38 + 3		38 + 2
Birth weight [g]	2015 (<3rd%)		2280 (<10th%)
Birth length [cm]	44 (<3rd%)		46 (<10th%)
Head circumference [cm]	30.5 (<3rd%)		32 (10–25th%)
Upper limb
Thumb hypoplasia	Right IIIa–b; left II		Right
Thumb aplasia	–		Left
Lower limb
Congenital hip dysplasia	–		+
Head and face
Microcephaly	−3SD^e^		−3SD
Microphthalmia	+		+
Growth
Small stature	−2SD		−4SD
GI system
Esophageal atresia	IIIb		–
Cardiac system
Congenital heart defect	–		VSD^f^
Other
Impaired hearing	+		–
Hypogammaglobulinemia	+ (transiently)		–
**Blood type**	0, Rh: positive		0, Rh: positive
**Bone marrow at diagnosis of ITP**	Normocellular, discreet dysplasia and atypia of all compartments; megakaryopoiesis numerically in the upper normal range, 10% of megakaryocytes mono-hypolobulated, no blasts		Moderately hypocellular, Blasts beneath 1%, megakaryocytes without dysplasia, but clearly reduced and with hyper-segmented nuclei suspicious of MDS transformation

	Patient 1		Patient 2
Complete blood counts (selected parameters)	Median (Min; Max)		Median (Min; Max)

Hemoglobin [g/dl]	12.4^j^ (9.90; 14.8)		12.5^j^ (11.4; 13.7)
Reticulocytes [T/l]	0.081^j^ (0.042; 0.145)		0.04^j^ (0.02; 0.09)
Mean corpuscular volume [fl]	81.2^j^ (76.8; 93.2)		86.8^j^ (84; 91.8)
White blood cells [/μl]	6640^j^ (3850; 12400)		4600^j^ (2800; 7200)
Lymphocytes [/μl]	3800^j^ (1700; 7300)		4080^j^ (2260; 7310)
Neutrophil granulocytes [/μl]	2210^j^ (670; 8800)		**1500** (1050; 3740)
**Cellular immune system**			
CD3 + T cells [/μl]	3555 | normal^j^		n.d.
CD3 + CD4 + T cells [/μl]	2663 | normal^j^		n.d.
CD3 + CD8 + T cells [/μl]	635 | normal^j^		n.d.
CD3–CD56 + NK cells [/μl]	**85 | moderately reduced**		n.d.
TRECs copies per 10^e^ CD3 + CD45+	60500 | normal^j^		n.d.
CD19 + B cells [/μl]	617 | normal^j^		n.d.
TCRa/b + CD4–CD8–CD3+	<2% of T cells | normal		n.d.
CD19 + CD27 + IgD+	>2% of B cells | normal		n.d.
CD19 + CD27 + IgD−	>2% of B cells | normal		n.d.

	Patient 1		Patient 2
Humoral immune system	Age: 9/12	1 8/12	6 1/12

IgG [mg/dL] | low/normal/high^g–i^	**177 | low^g^**	642 | normal^h^	1038 | normal^i^
IgG1 [mg/dL]	**121.49| low^g^**	498.28 | normal^h^	n.a.^a^
IgG2 [mg/dL]	**34.41 | low^g^**	69.5 | normal^h^	n.a.
IgG3 [mg/dL]	24.58 | normal^g^	73.83 | normal^h^	n.a.
IgG4 [mg/dL]	0.66 | normal^g^	0.39 | normal^h^	n.a.
IgA [mg/dL]	27.5 | normal^g^	54.3 | normal^h^	108 | normal^i^
IgM [mg/dL]	50.9 | normal^g^	90.6 | normal^h^	44 | normal^i^
IgE [IU/L]	4.2 | normal^g^	n.d.^b^	n.a.
Anti-diphtheria toxoid (DT) antibodies (Ab) [IU/L]	2.47 | normal^g^,^c^	2.37 | normal^h^,^c^	n.d.
Anti-tetanus toxoid (TT) Ab [IU/L]	4.15 | normal^g^,^c^	2.48 | normal^h^,^c^	n.d.
Anti-pneumococcus polysaccharide (PCP) Ab [mg/L]	42.96 |normal^g^,^c^	117.31 | normal^h^,^c^	n.d.
Anti-Haemophilus influenza B polysaccharide (HIB) Ab [mg/L]	3.16 | normal^g^,^c^	5.1 | normal^h^,^c^	n.d.

**Autoantibodies at diagnosis of ITP**			
Coombs test direct	Negative		Negative
Anti-platelet antibodies	Negative		**Positive: GpIIb/IIIa**
ANA	Negative		n.d.

	Patient 1		Patient 2

**Microbiological results at diagnosis of ITP**
Anti-Parvo B19 IgM, IgG	Negative		Negative
Anti-CMV IgM	**Positive**		Negative
Anti-CMV IgG	**Positive**		Negative
Anti-EBV IgM, IgG	Negative		Negative
Anti-HHV6 IgM	Negative		Negative
Anti-HHV6 IgG	Negative		**Positive**
HIV antigen and antibody	Negative		Negative
Anti-HCV	Negative		Negative
Anti-HBs IgG	Negative^c^		Negative
Anti-HBc IgG	Negative		Negative
Anti-VZV IgM	n.d.		Negative
Anti-VZV IgG	Negative		**Positive**
Anti-measles IgG	Positive^c^		Positive^c^
Stool
Rotavirus AG ELISA	n.d.		**Positive**
Norovirus AG ELISA	n.d.		Negative
Adenovirs AG ELISA	n.d.		Negative
Urine
CMV nucleic acid	**4–6 × 10^4^**		n.d.
	copies/mL^l^		
Blood plasma nucleic acid detection
PCR for CMV; EBV; AdV; HSV1,2; HHV6, ParvoB19; VZV; Enterovirus	Negative		n.d.

*^a^n.a., data not available or not done before IVIG administration*.

*^b^n.d., not done*.

*^c^After vaccination*.

*^e^SD, standard deviation*.

*^f^VSD, ventricular septal defect l*.

*^g–i^Age-adjusted reference values*.

*^g^IgG: 223–1099; IgG1: 140–620; IgG2: 41–130; IgG3: 11–85; IgG4: 0–0,8; IgA: 1–73; IgM: 8–100; DT IgG: >1; TT IgG: 0,02–3,12; PCP IgG: 0,9–93; HIB IgG: 0,08–9,2*.

*^h^IgG: 344–1180; IgG1: 220–720; IgG2: 50–180; IgG3:14–91; IgG4: 0–40,8; IgA: 2–98; IgM: 12–104; DT IgG: >1; TT IgG: 0,04–3,92; PCP IgG: 0,9–29,2; HIB IgG: 0,16–40,8*.

*^i^IgG: 411–1435; IgA: 34–214; IgM: 15–115*.

*^j^Normal according to age-dependent reference ranges*.

*^k^As described in the medical reports, precise data missing*.

*^l^At three occasions within 9 months*.

*Laboratory parameters in “bold” indicate pathologic results*.

The early manifestation of thrombocytopenia in FA, refractory to administration of platelet concentrates, warranted a more in-depth hematological evaluation. Apart from reduced platelet numbers, peripheral blood counts and erythrocyte indices remained normal during the period of observation (Table 1). Surprisingly, bone marrow examination did not show bone marrow failure or myelodysplastic syndrome (MDS; Table 1). In contrast, it revealed a marrow of normal cellularity, a megakaryocyte count in the upper normal range, and only mild dysplasia of all three cell lines, considered “normal for FA”. Neither signs of malignant infiltration and transformation to MDS were found, nor were any clonal chromosomal aberrations such as 1q+, 3q+, 7/7q−, 5/5q−, or trisomy 8 detectable. In the absence of a clinically apparent infection, PCR testing for HHV6, Parvovirus B19, CMV, and EBV from the bone marrow aspirate were performed with negative results. Assuming an immune-mediated mechanism IVIG were given, leading to an increase of platelets. Subsequently, the thrombocytopenia demonstrated a chronically persisting course with severe purpura, responding well to IVIG (Figure 1A). Anti-CMV IgG (analyzed before IVIG administration) and IgM were positive in blood sample, as was CMV nucleic acid in the urine (4–6 × 10^4^ copies/mL, Table 1), suggesting the presence of or recent recovery from a CMV infection, which represents a potential trigger of immune-mediated platelet destruction. The patient received a total of three platelet transfusions and seven IVIG infusions (5 with 0.8 g/kg body weight and two infusions with 0.5 g/kg body weight) within the first 6 months after presentation with ITP, but signs of bleeding (dry and wet purpura) and recurring thrombocytopenia persisted (Figure 1A). A short attempt of corticosteroid treatment with dexamethasone led to moderate response (platelet increase from 24,000 to 91,000/μl) but was terminated by the parents after 4 days because of inacceptable temper changes of the girl. Treatment with danazol, a synthetic androgen, was initiated at 5 mg/kg/day 5 months after the onset of thrombocytopenia, which slowly led to a satisfactory response with regards to bleeding tendency and was accompanied by a platelet count stabilization between 40,000 and 140,000/μl. Thus, the need of interventional IVIG treatment was substantially reduced (*n* = 2 per 6 months versus 7 per previous 6 months; Figure 1A). Side effects of danazol were weight gain (change of percentile from 10th to 25th within 9 months) and mood swings, both considered unspecific and tolerable for the patient by her family and treating physicians. Virilization or other endocrine abnormalities were not observed during the first 9 months of danazol treatment. The androgen dose could be reduced to 2 mg/kg after 6 months without change of platelet counts and will be further reduced if platelet counts remain stable. A recent follow-up bone marrow aspiration and trephine biopsy 1-year after the initial manifestation of thrombocytopenia showed no dynamics as compared to the previous analyses (not shown).

### Patient report 2

The second patient, a 7-year-old female was born on term with intrauterine growth retardation and multiple congenital abnormalities typical for FA. Furthermore, she failed to thrive (Table 1). A compound heterozygous *FANCD2* mutation confirmed the diagnosis of FA (compound heterozygous for a 3-bp-deletion and a missense substitution at codon 815) (6). Suddenly, at the age of 6 years severe thrombocytopenia (13,000/μl) was observed incidentally during a hospitalization for rotavirus gastroenteritis, while the red and white blood counts remained stable. Other (e.g., myelosuppressive) viral infections were ruled out serologically (Table 1). Similarly to the first patient, bone marrow aspiration and a trephine biopsy were performed immediately to verify suspected bone marrow failure, but no abnormalities other than FA-typical mild dysplasia, nor any cytogenetic aberrations were detected (Table 1). In the absence of myelosuppression or marrow failure, further evaluation was directed toward immune pathogenesis of the low platelet count. Positive antiplatelet antibodies against GpIIb/IIIa corroborated the diagnosis of ITP. Thrombocytopenia resolved after a single infusion of IVIG (Figure 1B), and platelet counts remained stable thereafter during 1 year after the initial presentation of ITP.

## Discussion

The simultaneous occurrence of two diagnostic entities, that have the potential to affect the platelet count through different, apparently even reciprocal pathogenetic mechanisms, namely ITP and FA, raised both diagnostic and therapeutic challenges. While in one patient with a *FANCA* mutation, the ITP started at 17 months of age and showed a chronically persisting course with severe purpura, that responded to IVIG and synthetic androgen therapy, the other patient (with *FANCD2* mutations) had a self-limited course that resolved after a single administration of IVIG. Other than FA-typical mild dysplasia, neither bone marrow abnormalities nor cytogenetic aberrations were detected. An association between platelet decline and anesthesia cannot be excluded in patient 1, because thrombopenia initially manifested shortly after gastroduodenoscopy in anesthesia. A complete blood count had not been analyzed immediately before general anesthesia.

Three possible interpretations for the concurrence of ITP and FA may be discussed, which are pathogenetically relevant and have implications for decisions of further treatment options. First, the unusual combination of FA and ITP might be interpreted as coincidental – presuming that ITP developed independently from the underlying FA. ITP occurs in 5 of 100,000 children per year, and FA is the most common inherited bone marrow failure syndrome (IBMFS), frequently underdiagnosed in patients without phenotypic abnormalities (7, 8). Additionally, ITP may be associated with CMV infection as present in the first patient (9, 10). Following this hypothesis, the therapeutic approach to thrombocytopenia is supposed to adhere to the general recommendations for treatment of ITP. Accordingly, both reported patients demonstrate satisfactory response to IVIG, known as first line therapeutic option for ITP (4). Although its effectiveness is largely attributed to immuno-modulating activity and considered an indirect evidence for an immune pathogenesis of an acute *de novo* thrombocytopenia, the exact mechanism of action of IVIG in ITP is still not entirely understood (11, 12). Interestingly, one study suggests a possible enhancement of thrombopoiesis by induction of endogenous thrombopoietin production following IVIG administration (12). Theoretically, in this case, platelet turnover might be positively influenced by IVIG both in ITP and FA. Currently, data for the efficacy of IVIG application in patients with FA are lacking. We did not treat patient 1 with thrombopoietin receptor agonists, because we generally aim to avoid hematopoietic growth factor administration in tumor predisposition syndromes. Patients with FA usually respond to treatment with androgens with an increase of erythropoiesis and, to a lesser extent, also thrombopoiesis (13–15). Likewise, a therapeutic efficacy of danazol is known in chronic ITP and other autoimmune hematologic disorders (Evans syndrome, autoimmune hemolytic anemia) (15–18). The exact mechanism of action of synthetic androgens is unknown, but immunomodulation is suspected to be involved (15).

Second, another explanation for the reported events could be that the underlying stem cell impairment facilitated or induced a subsequent immunologic reaction similar to that seen in refractory cytopenia of childhood and severe aplastic anemia (RCC/SAA) and thus, ITP might be considered “a feature of FA.” In this case, bone marrow transplantation would have to be considered an obvious therapeutic strategy like in MDS in FA (19). Thrombocytopenia is a well-known first manifestation of beginning bone marrow failure in FA (19), often associated with macrocytosis and progessive myelodysplasia. However, macrocytosis was absent as was myelosuppression in bone marrow analyses of both patients revealing normal cellularity, and even megakaryocytosis in the first patient. Additionally, none of the detected FA-associated gene mutations in our patients (*FANCA* and *FANCD2* mutations) are typically linked to early transformation to MDS or malignancy such as, e.g., *FANC IVS5* in Ashkenazi Jews, *FANCD1/BRCA2*, or *FANCN* mutations (20–24). Furthermore, patient 2 recovered soon after a single dose of IVIG, and patient 1 repeatedly showed normalization of platelet numbers upon IVIG, indicating a healthy regenerative capacity. Thus, MDS or bone marrow failure was unlikely to be the cause of thrombocytopenia seen in the presented FA patients aged 21 months and 6 years.

Third, a more complex, multifactorial etiopathogenensis of thrombocytopenia might be suspected in FA patients. A toxic effect of preceding anesthesia in patient 1 and detected CMV in patient 1 and Rotavirus in patient 2 or another undetected viral infection triggering immunologic events might have more pronounced effects in the setting of a “weak” bone marrow predisposed toward dysplasia than in healthy bone marrow. While MDS is usually unresponsive to IVIG, danazol is frequently used to treat cytopenia in FA and Dyskeratosis congenita (19, 25). Indirect evidence of immunoglobulin-supported remission of thrombocytopenia in patient 2 and the good response of platelet counts to IVIG (and later danazol) in patient 1, together with the only mild, benign bone marrow abnormalities described, support the hypothesis of an immune-mediated pathogenesis, but other contributing factors facilitating thrombocytopenia such as those mentioned cannot be excluded. Finally, in cannot be excluded that the administration of unmatched platelet concentrates in patient 1 (given three times within a short time frame after her first drop of platelet count) contributed to the immune pathogenesis and persistence of thrombocytopenia.

There is no evidence that complementation group or position or type of mutations in the present patients predisposed to ITP. Exome sequencing in a larger series of FA patients with ITP might unveil second gene variants as a basis for the association of these apparently coincidental clinical conditions.

## Conclusion

Thrombocytopenia is a common event during the natural course of FA. Because it is usually ascribed to bone marrow failure in FA, occurrence of ITP in those patients might be missed or misinterpreted. Our data show that acute and chronic ITP may occur in FA patients, and regular ITP-directed (IVIG) or “FA-adapted, ITP-directed” treatment options (such as danazol) proved successful. In contrast to current guidelines for the treatment of ITP (4), we chose the synthetic androgen danazol for the long-term treatment of chronically persisting ITP in a 2-year-old girl with FA and observed good response with tolerable side effects. With 15 months follow-up, we did not identify ITP as precursor of MDS in two pediatric FA patients.

## Author Contributions

MS, MB, RD, and CU designed and coordinated the study; DS performed genetic analyses; AK and MS wrote the final draft of the paper; SL and MS analyzed data and prepared tables and figures; SL, MS, AW, UB, SB, CU, and MB took care of the patients; all authors read and agreed to the final version of the manuscript.

## Conflict of Interest Statement

The authors declare that the research was conducted in the absence of any commercial or financial relationships that could be construed as a potential conflict of interest.
